# Butterfly Arch: A Device for Precise Controlling of the Upper Molars in Three Planes of Space

**Published:** 2013-05

**Authors:** Alireza Nikkerdar

**Affiliations:** ^1^Orthodontist, Private Practice, Tehran, Iran

**Keywords:** Trans-Palatal Arch; Tensile Strength; Anchorage Loss; Bracing Units

## Abstract

Intra-oral appliances such as transpalatal arch and Nance appliance fail to resist against forces that tend to loosen the anchorage. The infirmity arises due to the long lever arm and the mesial force that is perpendicular to the long axis of the appliance. The butterfly arch is presented here as an intra-oral appliance that withstands the mesially directed forces with a mechanism that puts strain on a stiff wire along its long axis. The unique shape of the butterfly arch is advantageous in maximum anchorage cases, cases in which arch width preservation is critical and cases with a vertical growth pattern. With the aid of the butterfly arch, clinical concerns such as patient cooperation, wearing extra-oral appliances, complicated mechanics in extraction cases and control of the arch length, arch width and vertical dimension would be greatly diminished.

## Introduction

Transpalatal arch (TPA) or palatal bar (PB) is an intra-oral tooth-borne appliance that is employed for numerous purposes in orthodontic practice. The usefulness of the appliance includes two basic categories: 1) active state and 2) passive state; in the active state the application of TPA involves a broad environment using different types and sizes of wires. Some functions are molar derotation, expansion, constriction, space gaining, correction of unilateral and bilateral crossbites and applying torque. 

Cetlin and Ten Hoeve used the palatal bar for correction of crossbites and severe rotations of maxillary first molars prior to the space gaining phase in non extraction treatment. They also indicated that the palatal bar is advantageous for gaining space adjacent to the first and second molars distal movements [1]. Ingervall et al. suggested a moment for molar derotation in the range of 30 mNm [2]. 

In correction of a unilateral crossbite, Ingervall and coworkers used a one-couple force system with a buccal root torque on the normal side and claimed that the expansive force in the system could open the mid-palatal suture in children [3]. Baldini and Luder studied the role of arch shape on the transverse effect of transpalatal arch during buccal root torque application. They found out that the most important factor in determination of moment-to-force ratio and the magnitude of expansive force was arch height [4]. In the passive state, the main goal is to preserve the initial position of the posterior teeth, especially the maxillary first molars in three planes of space. However, it has been traditionally used as an appliance for anchorage enhancement in the anteroposterior direction. Burstone introduced a special form of palatal arch that was made of 0.032"×0.032" stainless steel wire in special pre-torqued brackets (upper brackets with -12˚ torque) [5]. This intra-oral appliance is in accordance with “variable modulus orthodontics” concept in which different wires with different sizes such as 0.032"×0.032" TMA, 0.032"×0.032" SS and heavy round ones could be used [5, 6]. With the aid of finite element simulation, Kojima and Fukui showed that TPA had almost no effect on anchorage preservation against mesial movements [7]. 

**Fig 1:**
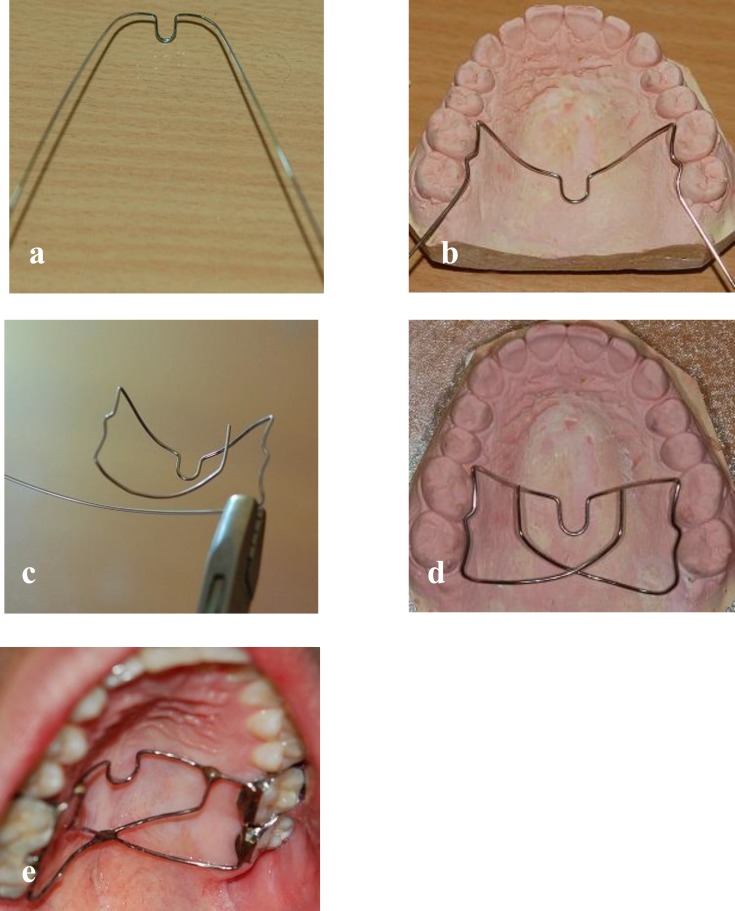
a to e, Stages for fabricating the butterfly arch. a, Bending the omega loop. b, Adjusting on the patient’s working cast. A toe-out bend is made at the contact area of the upper molars (arrow). c, The wire must follow the palatal contour, De La Rosa plier may be useful in this stage. d, The appliance is placed passively and adapted well to the teeth and palate. Refer to text for more information. e, The appliance with soldered points (arrows) is placed in the mouth successfully.

Zablocki and coworkers stated that TPA had no effect on either the anteroposterior or vertical position of the maxillary first molars during extraction treatment [8]. Wise and coworkers believed that impeding vertical alveolar growth of the maxillary first molars and tongue pressure against TPA surface were two primary mechanisms for vertical controlling of the upper posterior teeth [9]. With reference to the imperfections of intra-oral appliance (TPA and Nance appliance), to preserve anchorage and molar position, I introduce the butterfly arch (Fig 1) as an intra-oral appliance, extending from one side of the upper posterior teeth to the other side to maximize the efficacy of controlling the position of maxillary posterior teeth in all three planes of space.

***Fabrication***

The butterfly arch is consisted of a 0.036" stainless steel wire and four molar bands. Like conventional TPA for anchorage enhancement, there is an omega loop toward the distal at the midpoint of the upper first molars (Fig 1a). The loop should be as small as possible. Therefore, only 5 to 7 mm of the diameter is appropriate. Then, the wire should be bent out at the contact area of the first and second molars (Fig 1b). Later on, the wire should be extended 1 mm distally to leave the second molar band completely and must be bent back with a mild curve toward the opposite side (black arrows in Fig 1d). Another bend (approximately 110˚) should be performed near the contact area of the first and second molars at the contradictory side (arrowhead in Fig. 1d) going forward to the mid-point of the distance between the mesio-palatal cusp of the first molar and the omega loop. The same performance should be carried out for the opposite side. The intersection point must be placed along the omega loop at the mid-palatal region and should form a box shape soldering area (Fig 1e). 

**Fig 2:**
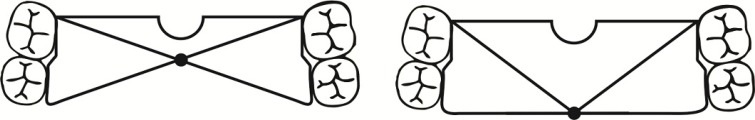
Other designs of the butterfly arch

This type of configuration provides a rigid structure that is especially pertinent in posterior critical anchorage cases. Later on, the wire should be soldered at the mesio-palatal line angle of the first and second molar bands. However, the amount of soldering material is slightly more than what is essential for fabricating conventional TPA and it should be extended slightly along the palatal surfaces of the upper first and second molar bands (Fig 1e). When vertical control of the upper molars is essential, the wires must be kept 4 to 5 mm away from the palatal mucosa. At the buccal side, if initial alignment is not established, we can hold the first and second molars together throughout passive ligation or segmented technique. Another way is to insert a continuous wire with compensatory bends. Other designs of the butterfly arch are depicted in fig 2 but my experience over three years suggests the one that is shown in Fig 1.

**Fig 3:**
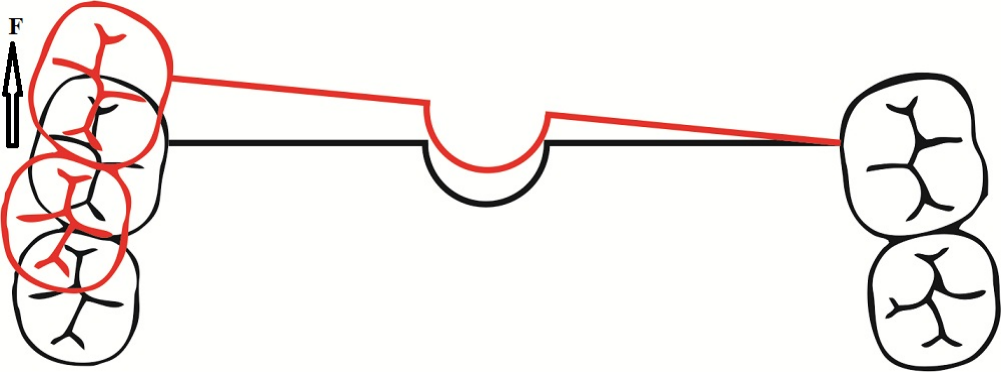
Long lever arm of TPA could not resist against forces that are perpendicular to its long axis. F, Mesial force dashed line, lever arm

***Force system and biomechanics***

The biomechanics of transpalatal arch is well understood [10]. The failure of TPA to preserve the anchorage lies beneath the inability to resist against forces that apply perpendicular to its long axis. If a beam with a small cross-section attaches firmly to a point and if a force with slight magnitude applies on the other side, the beam rotates simply around the point of attachment (Fig 3). 

**Fig 4:**
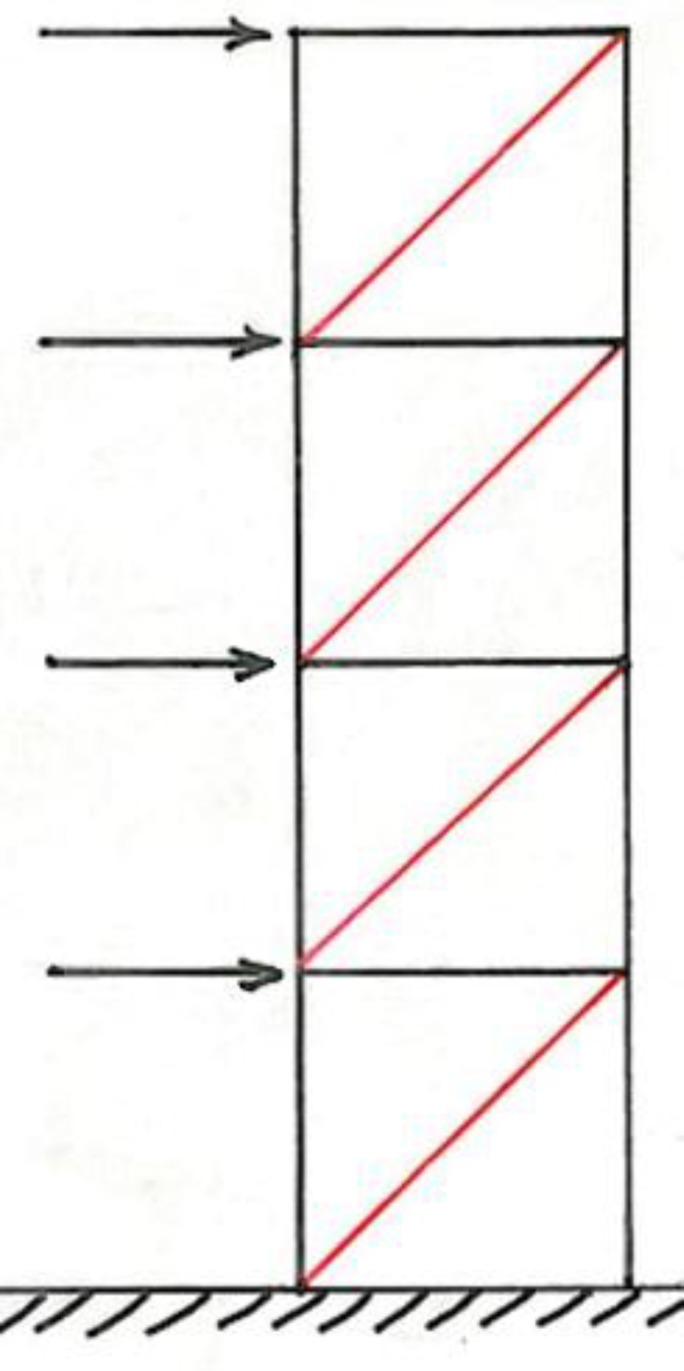
Bracing systems

In an earthquake resistant structure, the bracing element, which is a beam with a small cross-section, is set to counteract horizontal forces of earthquake (Fig 4).

The reason lies beneath this fact that when horizontal stress exerts (black arrows in Fig 4), the tension raises in the bracing unit (red fractions in Fig 4). 

It is hard to overcome the tensile strength of the beam even with a small cross-section. Therefore, these elements strengthen the total resistance of the building against the horizontal forces of earthquake.

**Fig 5:**
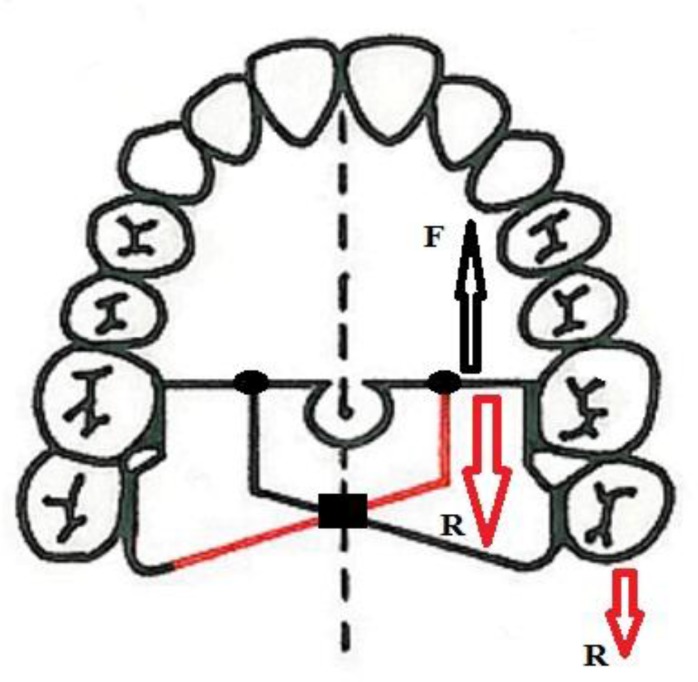
Butterfly arch. Mesially directed force (F) must prevail over two main resistance areas (red arrows). Soldering points (blue arrows) provide additional retention areas and increase the total resistance of the appliance. Refer to text for more information.

Butterfly arch design is based on the same principle. In fig 5, the mesial force (black arrow), which appears due to retraction of anterior teeth, tends to move the teeth of the anchorage unit mesially. However, the red fraction withstands this type of load. A difference between the red fraction of the butterfly arch and the one in the bracing system does exist. 

The mesial force must undo the bend (110˚) of this fraction and then, must prevail over the tensile strength of the wire along its long axis that is too large to overcome. Furthermore, soldering points provide additional retention areas (blue arrows in Fig 5) that would enhance the total retention of the apparatus and reduce the anchorage loss. The unique configuration of the appliance provides a wide surface which contributes a good control on the vertical dimension by using tongue function and pressure. With regard to the high rigidity of the appliance due to stiff stainless steel wire and short segments, the transverse dimension could be well-preserved as well. 

## Discussion

Clinical usage of butterfly arch involves numerous applications. In maximum anchorage cases such as class I crowding, class I bimaxillary protrusion and many of class II cases, the butterfly arch preserves the arch length and arch width in anteroposterior and transverse directions, respectively. The arch resists well against the “rolling-in” tendency and “rowboat” effect during space closure. Control of the arch width is crucial in treating some types of asymmetries such as correction of occlusal cants and midlines.

 Leveling of high canines is another challenge which needs to be taken into special care in the matter of transverse dimension. In high angle cases, control of the vertical dimension is prominent and requires special care during all stages of treatment. Elements of the appliance provide an extensive platform for the tongue to exert intrusive force upon upper posterior teeth. After eruption of the second molars, the butterfly arch could be used for children with a vertical growth pattern and mandibular deficiency with or without some kind of class II functional appliances. In these cases, the burden of wearing headgear would be eliminated. At first, fabrication seems to be difficult and time-consuming, but a skilled wire- bender needs a few minutes to shape and solder the appliance meticulously. In spite of its complex appearance, the appliance is well tolerated by the patient and fracture is not common.

## CONCLUSION

Butterfly arch provides a good control on the position of the upper posterior teeth in all three planes of the space. For acting well, the appliance should be fabricated precisely and symmetrically and must be placed passively into the mouth. Although the butterfly arch has many advantages, more study and more practice should be carried out in the future in the field of various functions of the appliance. 
